# 66534 Evaluation plans for a summer child nutrition assistance program to better understand translation of policy to community health

**DOI:** 10.1017/cts.2021.746

**Published:** 2021-03-30

**Authors:** Jiwoo Lee, Jayne A. Fulkerson, Lisa J. Harnack, Weihua Guan

**Affiliations:** 1 School of Nursing, University of Minnesota; 2 Division of Epidemiology and Community Health, School of Public Health, University of Minnesota; 3 Division of Biostatistics, School of Public Health, University of Minnesota

## Abstract

ABSTRACT IMPACT: Study findings can guide improvements of the Summer Food Service Program to maximize the program’s desired effects on child summer nutrition and related health outcomes. OBJECTIVES/GOALS: The Summer Food Service Program (SFSP) addresses food insecurity during summer months. Project specific aims are to: 1. Describe characteristics of children participating in the SFSP. 2. Determine the nutritional quality of the SFSP foods. 3. Evaluate changes in children’s food insecurity, diet quality, and body mass index by SFSP participation. METHODS/STUDY POPULATION: A single group, prospective, staggered cohort design will be used for the proposed study. Two cohorts of 30 (N=60) elementary students and their parents will be recruited during the 2021-22 and 2022-23 school year. Each participant will complete a measurement session at three time-points: Baseline (spring), Post-Program (program end), and Follow-Up (following spring). Parents will complete an online survey about household food insecurity and family socio-demographic characteristics. Children will complete three 24-hour dietary recall interviews, and their heights, weight and percent body fat will be measured. The menus of at least ten SFSP sites will be analyzed to determine the nutritional adequacy of the site menus by using the Healthy Eating Index-2015. RESULTS/ANTICIPATED RESULTS: Study hypotheses are as followed: Aim 1. Not all of the children participating in the SFSP are from food-insecure or low-income households. Aim 2. Meals served at the SFSP will be higher in sugar and fat and lower in fruits and vegetables compared to recommendations in the 2015-2020 Dietary Guidelines for Americans. Additionally, the Healthy Eating Index-2015 score of the SFSP menus will be lower than that of the National School Lunch Program menus. Aim 3. Consistent SFSP participation will have a positive effect on reducing food insecurity, but not on increasing diet quality and reducing body mass index and percent body fat in children. DISCUSSION/SIGNIFICANCE OF FINDINGS: Program user information will determine if the program is reaching the target audience. Program managers will utilize menu analysis results to improve their menu nutritional quality. Changes in food insecurity, diet quality and anthropometric measures will inform whether the program needs to be improved to prevent any untoward excess weight gain.

